# Ferritin Is Secreted from Primary Cultured Astrocyte in Response to Iron Treatment via TRPML1-Mediated Exocytosis

**DOI:** 10.3390/cells12212519

**Published:** 2023-10-25

**Authors:** Xiaoqi Yu, Zhixin Xiao, Junxia Xie, Huamin Xu

**Affiliations:** 1 Shandong Provincial Key Laboratory of Pathogenesis and Prevention of Neurological Disorders, Department of Physiology, School of Basic Medicine, Qingdao University, Qingdao 266071, China; 2 Institute of Brain Science and Disease, Qingdao University, Qingdao 266071, China

**Keywords:** Parkinson’s disease, ferritin, astrocytes, iron, transient receptor potential mucolipin 1

## Abstract

Impaired iron homeostasis has been proven to be one of the critical contributors to the pathology of Parkinson’s disease (PD). Ferritin is considered an intracellular protein responsible for storing cytosolic iron. Recent studies have found that ferritin can be secreted from cells independent of the classical endoplasmic reticulum–Golgi system. However, the precise mechanisms underlying the secretion of ferritin in the brain were not elucidated. In the present study, we demonstrated that the primary cultured astrocytes do have the ability to secrete ferritin, which is enhanced by iron treatment. Increased ferritin secretion was accompanied by increased protein expression of ferritin response to iron stimulation. Further study showed that iron-induced expression and secretion of ferritin could be inhibited by CQ or 3-MA pretreatment. In addition, the knockdown of transient receptor potential mucolipin 1 (TRPML1) antagonized iron-induced ferritin secretion, accompanied by further increased intracellular protein levels of ferritin. Further study demonstrated that ferritin colocalized with LAMP1 in iron-treated astrocytes. On the contrary, ras-associated protein 27a (Rab27a) knockdown further enhanced iron-induced ferritin secretion and decreased intracellular protein levels of ferritin. Furthermore, we also showed that the secretory autophagy protein tripartite motif containing 16 (TRIM16) and sec22b decreased in iron-treated astrocytes. These results suggested that astrocytes might secrete ferritin via TRPML1-mediated exocytosis. This provides new evidence for the mechanisms underlying the secretion of ferritin in primary cultured astrocytes under a high iron environment.

## 1. Introduction

Parkinson’s disease (PD) is a common neurodegenerative disease. The pathological characteristics of PD is the degeneration of dopamine (DA) neurons in the substantia nigra pars compacta (SNpc), resulting in a decrease in DA in the striatum [1,2,3,4]. This leads to a series of motor symptoms, including dyskinesia, tremor, and stiffness. Although the etiology of PD is not yet fully understood, studies have shown that mitochondrial dysfunction, oxidative stress, misfolded α-synuclein, and the excessive deposition of iron might induce the occurrence of PD [5,6,7]. More and more evidence showed that abnormal nigral iron deposition was one of the key contributors to the etiology of PD [5,8,9,10,11]. Iron deposits were found in the substantia nigra (SN) of PD patients and PD animal models, and increased nigral iron levels were reported to be closely related to the progress of PD [5]. Excess iron can produce reactive oxygen species (ROS) through the Fenton reaction, promote the accumulation of α-synuclein, and accelerate the degeneration of DA neurons [12,13,14,15,16,17].

Iron is the most abundant trace element in mammals and participates in a variety of physiological processes in the brain, including myelination, neurotransmitter synthesis, oxidative phosphorylation, etc. [13,18]. In the brain, iron is mainly combined with ferritin, which is a key protein for iron storage in the brain [19,20]. Ferritin contains 24 subunits, composed of H-ferritin and L-ferritin. H-ferritin has ferroxidase activity, which converts Fe^2+^ to Fe^3+^ and stores it in the iron core of ferritin. L-ferritin is mainly responsible for promoting iron deposition and ultimately storing iron in a soluble, non-toxic, and bioavailable form [21,22]. Studies have confirmed that the ferritin level in the SN of PD patients is lower than that of the normal control, suggesting that the ferritin load in the SN may be higher than that of the normal control [23,24,25]. The excess iron cannot be stored in a non-toxic and soluble form, which makes DA neurons of the SN more susceptible to oxidative stress damage and aggravates the pathological process of PD.

In most eukaryotes, ferritin has been classically considered as an intracellular iron storage protein. Recent studies have found that ferritin can exist outside the cell as a secreted protein, suggesting that ferritin may be an important extracellular protein to regulate iron homeostasis [26]. Studies have found that in insects, ferritin is a classic secreted protein that plays an important role in the distribution of iron in the system [26]. Most eukaryotic secretory proteins have an N-terminal signal peptide, which enables them to enter the endoplasmic reticulum (ER) and secrete the protein outside the cell through the classical secretory pathway through the Golgi apparatus [27]. Ferritin in mammals lacks the N-terminal signal peptide and cannot be secreted through the classical secretory pathway. It has been reported that non-classical lysosomal secretion might be involved in ferritin secretion from cells [26]. Studies have also found that macrophages in mice can transfer ferritin between cells through the non-classical lysosomal secretion pathway and exosomal pathway [28]. However, the mechanisms underlying ferritin secretion in the brain, especially in the condition of high iron levels, are not fully understood.

Studies have confirmed that ferritin is expressed in oligodendrocytes, microglia, and astrocytes in the brain [29]. Among them, astrocytes are the most abundant in the brain. They provide metabolic support to neurons and regulate synaptic transmission [30]. Astrocytes also play an important role in the regulation of iron homeostasis in the brain and protect other cells in the brain against iron-mediated oxidation. In a proteomics study, it was found that ferritin can be secreted by astrocytes, suggesting that astrocytes might play a key role in the secretion of ferritin in the brain [31]. Therefore, in this study, primary cultured astrocytes of the midbrain were used to explore the possible mechanism underlying ferritin secretion from astrocyte in response to iron treatment. This will provide an important theoretical basis for elucidating the secretion mechanism of ferritin induced by iron in astrocytes and provide a new target to regulate iron homeostasis in PD.

## 2. Materials and Methods

### 2.1. Cell Cultures and Treatments

All procedures were approved by the Animal Ethics and Experimentation Committee of the Qingdao University. Primary astrocyte culture was performed according to a previous study [32,33]. Midbrains of Newborn Wistar rats within 24 h after birth were isolated under stereomicroscopy. After washing with PBS, the tissues were mechanically dissociated and filtered through a 100 µm mesh to obtain a single-cell suspension. After centrifugation at 1000 rpm for 5 min, the cells were resuspended and then cultured in 150 cm^2^ culture flasks at a density of five mouse brains per 75 cm^2^ in incubator at 37 °C with 5% CO_2_. The cell medium was changed every 3 days. After 8–10 days, astrocytes were harvested by oscillating at 200 rpm for 18–20 h at 37 °C to remove microglia and oligodendrocyte precursor cells. The attached astrocytes were trypsinized by 0.25% trypsin (Hyclone, Logan, UT, USA), and then the culture medium containing 5% fetal bovine serum (FBS) (Gibco, Grand Island, NY, USA) was added to terminate the reaction. The cell mixture was transferred to a 50 mL centrifuge tube, centrifuged at 1000 rpm for 5 min, and the supernatant was removed. Cells were then resuspended in medium containing 5% FBS and 1% antibodies (Solarbio, Beijing, China) and cultured on plates or coverslips for the following experiments.

Astrocytes were seeded at a density of 1.5 × 10^5^/mL per well in 6-well plates. After pretreatment with 30 μM lysosomal inhibitor chloroquine (CQ) [34] or 2.5 mM autophagy prophase inhibitor 3-methyladenine (3-MA) [35] for 30 min, astrocytes were incubated with 100 μM ferric ammonium citrate (FAC) for the following 24 h. Then, astrocytes were collected to detect the protein expression of ferritin, and the supernatant was collected to detect the level of extracellular L-ferritin and iron.

### 2.2. Detection of Cell Viability by LDH Assay Kit

According to the manufacturer’s instructions, LDH assay kit was used to detect the effect of iron on cell viability of astrocytes by measuring the LDH release in the supernatants (Jiancheng Bioengineering Technology, Nanjing, China).

### 2.3. Immunofluorescence Staining

The immunofluorescence staining was performed as described in the literature [36]. Astrocytes were cultured on glass coverslips. Then, cells were fixed with 4% paraformaldehyde for 15 min. After washing with 1 × PBS three times and blocking with PBST containing 5% goat serum for 2 h, cells were stained with the primary antibodies of GFAP (CST, Boston, MA, USA, 1:50), L-ferritin (Abcam, Cambridge, UK, 1:50), and LAMP-1 (Abcam, Cambridge, UK, 1:50) overnight at 4 °C and then labeled with a secondary goat anti-rabbit Alexa Fluor^®^-488-conjugated antibody (Thermos Fisher Scientific, Waltham, MA, USA, 1:500) or goat anti-mouse Alexa Fluor^TM^ 555 (Thermos Fisher Scientific, Waltham, MA, USA, 1:500). Digital pathology section system (OLYMPUS, Tokyo, Japan) was used to collect images.

### 2.4. Protein Extraction

RIPA lysis buffer (Beyotime, Shanghai, China) containing protease inhibitors was used to extract total protein from astrocytes. After lysing on ice for 30 min, cells were scraped off the culture plate with a cell scraper and transferred to a 1.5 mL centrifuge tube. After centrifuging at 12,000 rpm for 30 min at 4 °C, the supernatant was collected. Bicinchoninic acid (BCA) kit (CWBIO, Taizhou, Jiangsu, China) was used to determine protein concentration.

### 2.5. Western Blots

Western blots were performed as described previously [37]. The total 20 μg of protein was separated on 10% sodium dodecyl sulfate-polyacrylamide gel electrophoresis (SDS-PAGE) and transferred to a polyvinylidene fluoride (PVDF) membrane. Then, 5% non-fat dry milk in Tris-buffered saline-tween-20 (TBST) was used to block non-specific reactivity. The PVDF membranes were incubated with different antibodies: L-ferritin (Abcam, Cambridge, UK, 1:1000), H-ferritin (Abcam, Cambridge, UK, 1:1000), Rab27a (R&D System, Minnesofa, MN, USA, 1:2000), transient receptor potential mucolipin 1 (TRPML1) (Novus Biologicals, Littleton, CO, USA, 1:1000), tripartite motif containing 16 (TRIM16) (BEHYL, Waltham, MA, USA, 1:1000), sec22b (Abcam, Cambridge, UK, 1:1000), iron regulatory protein 1 (IRP1) (Abcam, Cambridge, UK, 1:2000), and anti-β-actin (Bioss, Beijing, China, 1:10,000) at 4 °C overnight. Then, the membranes were incubated with horseradish peroxidase (HRP)-conjugated secondary antibodies (1:10,000) for 1 h at room temperature. The enhanced chemiluminescence (ECL) Western blotting detection system (Millipore Corp., Billerica, MA, USA) was used to detect the bands, and Image J 1.8.0 software (NIH Image, Bethesda, MD, USA) was used to analyze the bands.

### 2.6. Iron Assay Kit

Iron concentration was measured by iron assay kit (Abcam, Cambridge, UK) according to the manufacturer’s instructions as described by our previous study [33]. Cell culture medium was collected and centrifuged at 4 °C, 12,000 rpm for 10 min. Then, 100 µL of the standard dilutions or samples was added to 96-well plate. Add 5 μL iron reducer to each standard well and 5 µL iron reducer (for total iron) or assay buffer (for Fe^2+^) to each sample. After incubation for 30 min at 37 °C, 100 µL of iron probe was added to each well and incubated for 60 min at 37 °C in the darkness. The absorbance at 593 nm was measured using a microplate reader (SpectraMax M5, Molecular Devices, San Jose, CA, USA). After subtracting the average absorbance value of the blank from standard and sample readings, the optimal smooth curve was drawn based on the corrected absorbance values of each standard to construct the standard curve. The contents of total iron and Fe^2+^ in the samples can be obtained from the standard curve. Finally, the content of ferric iron (Fe^3+^) in the samples can be calculated as total iron (Fe^2+^ + Fe^3+^) − ferrous iron (Fe^2+^).

### 2.7. Enzyme-Linked Immunosorbent Assay (ELISA)

ELISA kit of L-ferritin (Abcam, Cambridge, UK) was used to detect the levels of L-ferritin in the astrocyte supernatant. A total of 100 µL of each sample or standard was added to a 96-well plate and incubated at room temperature for 60 min. The samples were removed and washed with washing solution 3 times. Then, 100 µL of 1 × enzyme-antibody conjugates was added and incubated at room temperature for 10 min. After washing with 1 × wash buffer three times, 100 µL of the TMB substrate solution was added to each well and then incubated for 10 min in the darkness. Finally, 100 µL of stop solution was added to each well to stop the reaction. The optical density of each well was measured immediately using a microplate reader (SpectraMax M5, Molecular Devices, San Jose, CA, USA) at 450 nm.

### 2.8. Knockdown of Rab27a and TRPML1

For silencing of the Rab27a or Mcoln1 gene in primary cultured astrocytes, lentiviruses carrying short hairpin RNA (shRNA) targeting Rab27a (pSLenti-U6-Rab27a shRNA-CMV-EGFP-F2A-Puro-WPRE) or Mcoln1 (pSLenti-U6-Mcoln1 shRNA-CMV-EGFP-F2A-Puro-WPRE) were purchased from OBiO (Shanghai, China) and used in this study. Lentivirus-carrying control scrambled shRNA was used as a control. Briefly, astrocytes were seeded in six-well plates and were infected with pSLenti-U6-Rab27a shRNA-CMV-EGFP-F2A-Puro-WPRE or pSLenti-U6-Mcoln1 shRNA-CMV-EGFP-F2A-Puro-WPRE for 72 h. At the end of the infection, cells were harvested for mRNA and protein analysis to determine the knockdown effect by quantitative real-time PCR (RT-PCR) and Western blots, respectively.

### 2.9. Real-Time PCR

Real-time PCR was performed as described before [38]. Total RNA was extracted by trizol reagent (Invitrogen, Carlsbad, CA, USA). Reverse transcription kit (abm, Hangzhou, Zhejiang, China) was used, and RT-PCR was carried out using an Eppendorf real-time fluorescent quantitative PCR with the SYBgreen PCR kit (abm, Zhejiang, China). The primers for Rab27a were as follows: forward, 5′-ACCTCGGGCATCCATCTGTAACG-3′; reverse, 5′-TTCCCCCACCCCCAAACTCA-3′. The primers for TRPML1 were as follows: forward, 5′-AAACACCCCAGTGTCTCCAG-3′; reverse, 5′-GAATGACACCGACCCAGACT-3′. PCR reactions were run at 94 °C for 5 min, followed by 35 cycles of denaturation at 94 °C for 30 sec, annealing at 58 °C for 30 s, and elongation at 72 °C for 1 min; then final elongation was performed at 72 °C for 5 min. The mRNA level was normalized to GADPH and calculated with the 2^−∆∆Ct^ method.

### 2.10. Statistical Analysis

Statistical analysis was performed using GraphPad Prism 7.0. Student’s *t*-test was used to compare two groups. One-way ANOVA followed by Bonferroni post hoc test was performed to compare more than two groups. Data are presented as the mean ± SEM. Difference was considered to be statistically significant at *p* < 0.05.

## 3. Results

### 3.1. Iron Treatment Enhanced the Expression of Ferritin via IRP1 in Astrocytes

**Figure 1:**
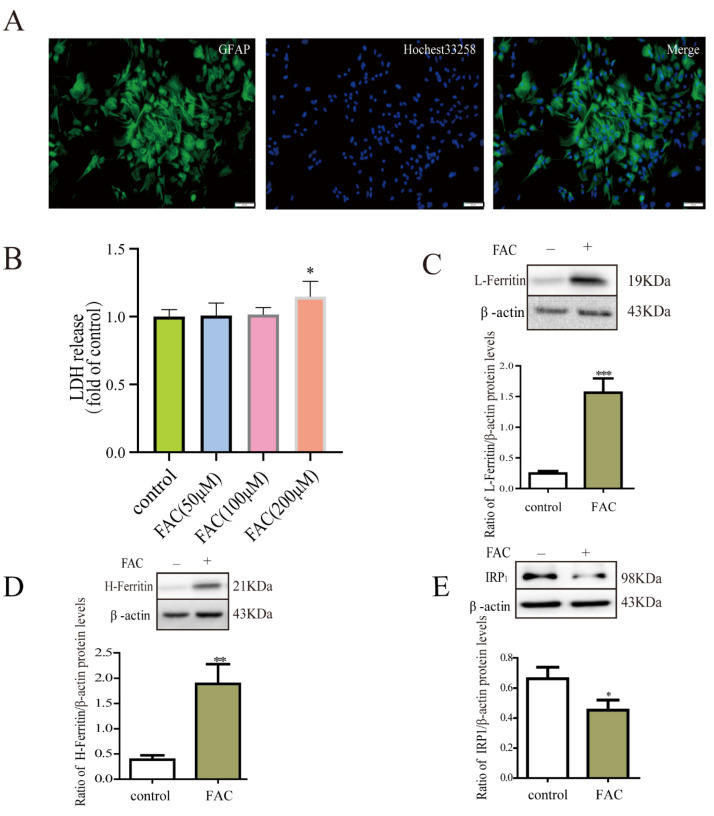
Effects of FAC on LDH release and the protein expression of ferritin and IRP_1_ in astrocytes. (**A**) Images of immunofluorescent staining with antibody against GFAP as characteristic marker of astrocytes. Scale bar: 20 µm. (**B**) LDH release of primary astrocytes incubated with 50 μM FAC, 100 μM FAC, or 200 μM FAC for 24 h. LDH release of primary astrocytes increased after 200 μM FAC treatment. n = 8. (**C**–**E**) After treatment with 100 μM FAC for 24 h, the expression of L-ferritin (**C**), H-ferritin (**D**), and IRP_1_ (**E**) was detected using Western blots. The protein expression of L-ferritin and H-ferritin increased, and the expression of IRP1 decreased after FAC treatment compared with the control. Data are presented as the mean ± SEM, n = 8, * *p* < 0.05, ** *p* < 0.01, and *** *p* < 0.001, compared with the control.

GFAP is a widely used marker of astrocytes. Our results showed that the purity of astrocytes was over 98% (Figure 1A). The cell viability of astrocytes was not affected by 100 μM FAC based on the LDH assay (Figure 1B). Then, Western blot was used to detect the protein expression of L-ferritin, H-ferritin, and IRP_1_ in the primary cultured astrocytes after FAC treatment. Results demonstrated that the protein expression of L-ferritin and H-ferritin increased, and the expression of IRP1 decreased in astrocytes after iron treatment compared to the control (Figure 1C–E). This suggested that the increase of ferritin in response to iron treatment was dependent on the IRP/IPE system.

### 3.2. Iron Treatment Increased Extracellular Iron and Enhanced Secretion of Ferritin from Astrocytes

**Figure 2:**
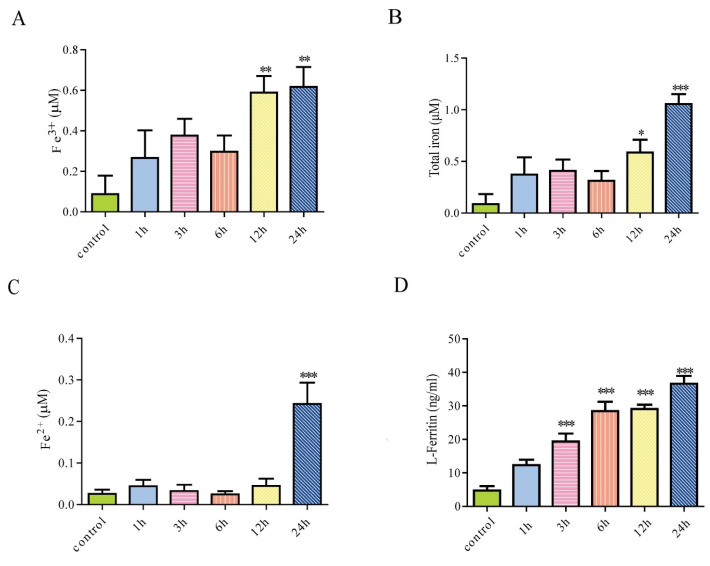
The effect of FAC treatment on extracellular iron level and ferritin secretion of astrocytes in different incubation times. (**A**–**C**) After treatment with 100 μM FAC for 1 h, 3 h, 6 h, 12 h, and 24 h, the content of Fe^3+^ (**A**), total iron (**B**), and Fe^2+^ (**C**) in the cell supernatant was detected. (**D**) After treatment with 100 μM FAC for 1 h, 3 h, 6 h, 12 h, and 24 h, the level of L-ferritin in the cell supernatant was detected using ELISA kit. Data are presented as the mean ± SEM, n = 6,* *p* < 0.05, ** *p* < 0.01, and *** *p* < 0.001, compared with the control.

We detected the concentration of Fe^2+^, Fe^3+^, and total iron in the cell culture medium of astrocytes after treatment with 100 μM FAC for 1 h, 3 h, 6 h, 12 h, and 24 h. Results showed that Fe^3+^ and total iron content in the cell supernatant was increased after FAC treatment for 12 h and 24 h. The content of Fe^2+^ in the cell supernatant was increased when treated with FAC for 24 h (Figure 2A–C). To further investigate whether primary cultured astrocytes can secrete ferritin, an ELISA kit was used to detect the level of ferritin in the cell supernatant. Results showed that the secretion of L-ferritin in primary cultured astrocytes increased significantly after iron treatment for 3 h, 6 h, 12 h, or 24 h compared to the control (Figure 2D). The cell viability of astrocytes was not affected by 100 μM FAC, indicating that L-ferritin in the media of astrocytes is the result of secretion but not the result of cell lysis. Ferritin is a heteropolymer of H and L subunits. This indicates that the primary cultured astrocytes could secrete ferritin, which could be enhanced by iron treatment.

### 3.3. Lysosomal Secretory Pathway Might Be Involved in Iron-Induced Secretion of Ferritin

**Figure 3:**
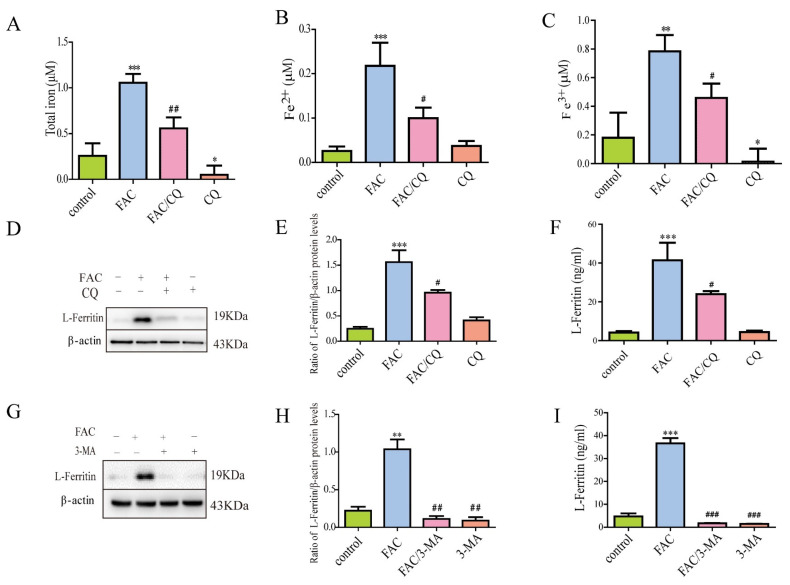
CQ or 3-MA inhibited iron-induced ferritin secretion. (**A**–**C**) After pretreatment with 30 μM CQ for 30 min, cells were incubated with 100 μM FAC for 24 h, and the content of total iron (**A**), Fe^2+^ (**B**), and Fe^3+^ (**C**) in the cell supernatant was detected. (**D**,**E**) CQ inhibited iron-induced increase in the expression of L-ferritin. Western blots were used to detect the protein expression of L-ferritin. (**F**) CQ inhibited iron-induced L-ferritin secretion. ELISA kit was used to detect the level of L-ferritin in the cell supernatant of astrocytes. (**G**,**H**) 3-MA inhibited iron-induced increase in the expression of L-ferritin. (**I**) 3-MA inhibited iron-induced L-ferritin secretion. Data are presented as the mean ± SEM, n = 6, * *p* < 0.05, ** *p* < 0.01, and *** *p* < 0.001, compared with control; ^#^ *p* < 0.05, ^##^ *p* < 0.01, and ^###^ *p* < 0.001, compared with FAC treatment.

Studies have reported that the non-classical lysosomal secretion might be involved in the secretion of ferritin in macrophages [26]. In order to explore the possible mechanisms underlying the iron-induced secretion of ferritin from primary cultured astrocytes, a lysosomal inhibitor chloroquine (CQ) was chosen to inhibit autophagosome–lysosome fusion. Then, we detected the extracellular iron content and the expression and secretion of L-ferritin in astrocytes. Results showed that CQ pretreatment inhibited the iron-induced increase in total iron, Fe^2+^, and Fe^3+^ in the supernatant of astrocytes, compared with the control group (Figure 3A–C). Then, we further detected the expression of L-ferritin in astrocytes using Western blots. Results showed that FAC treatment increased the expression of L-ferritin significantly in primary cultured astrocytes, which could be antagonized by CQ (Figure 3D,E). We further investigated whether CQ affected the secretion of L-ferritin in iron-treatment astrocytes. Results showed that CQ pretreatment significantly inhibited the iron-induced secretion of L-ferritin (Figure 3F).

Furthermore, autophagy prophase inhibitor 3-MA was used to inhibit autophagy initiation, and then the expression and secretion of L-ferritin in astrocytes were detected. Results demonstrated that 3-MA significantly inhibited the iron-induced increase in protein expression and secretion of L-ferritin in primary cultured astrocytes (Figure 3G–I).

### 3.4. Ferritin Colocalized with LAMP1 in Iron-Loaded Astrocytes

**Figure 4:**
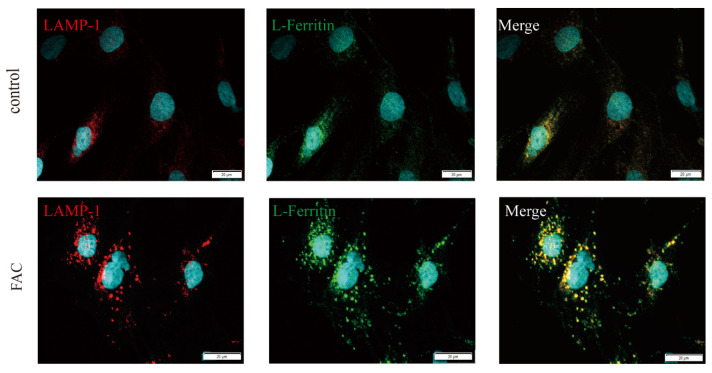
Ferritin colocalized with LAMP-1 in astrocytes. Co-immunostaining for L-ferritin (green) and LAMP-1 (red) in astrocytes (**top**) and iron-loaded astrocytes (**bottom**). Scale bar 20 µm.

LAMP-1 is a highly glycosylated protein in lysosome membranes and is used as a lysosomal-specific marker [39]. In this study, to further confirm whether ferritin accumulates in lysosomes of astrocytes, subcellular localization of L-ferritin was detected using immunolocalization. The punctate distribution of intracellular L-ferritin and colocalization of L-ferritin with LAMP1 was observed in astrocytes. In addition, increased fluorescence of L-ferritin and LAMP1 was observed in iron-treated astrocytes (Figure 4). And the size of LAMP1-positive lysosomes was also strongly increased by iron treatment. This suggests that ferritin might be delivered to lysosomes, but degradation of ferritin might be impaired by iron treatment. This indicated that lysosomes might be involved in the iron-induced secretion of ferritin in astrocytes.

### 3.5. Lentivirus-Mediated TRPML1 Gene Knockdown Antagonized Iron-Induced Ferritin Secretion

**Figure 5:**
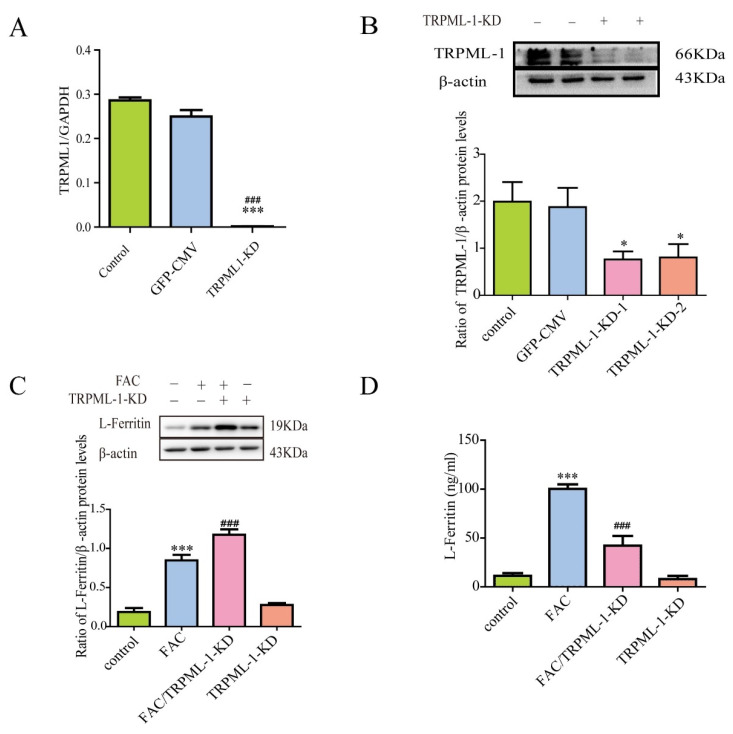
TRPML-1 inhibited iron-induced ferritin secretion of astrocytes. (**A**,**B**) TRPML-1 knockdown decreased the mRNA and protein levels of TRPML-1. The efficiency of TRPML-1 knockdown in astrocytes was evaluated by qPCR (**A**) and Western blots (**B**). n = 10, * *p* < 0.05, and *** *p* < 0.001, compared with control; ^###^ *p* < 0.001, compared with GFP-CMV. (**C**,**D**) TRPML-1 knockdown enhanced iron-induced increase of intracellular L-ferritin protein levels and inhibited iron-induced L-ferritin secretion. Western blots were used to detect the intracellular protein level of L-ferritin (**C**); ELISA kit was used to detect the extracellular level of L-ferritin (**D**). Data are presented as the mean ± SEM, n = 6, *** *p* < 0.001, compared with control; ^###^ *p* < 0.001, compared with FAC.

TRPML1 is a cation channel and exists in the endosomal and lysosomal membranes. It has been demonstrated that TRPML1 mediates the release of lysosomal Ca^2+^ [40], which is involved in late endosome and lysosome fusion and lysosomal exocytosis. In order to further clarify the underlying mechanisms of ferritin secretion in response to iron treatment, we designed and synthesized a lentivirus that could knock down the TRPML1 gene. Then, we detected the expression of TRPML1 in astrocytes and the level of L-ferritin secreted from astrocytes using WB and ELISA. Our results showed that TRPML1 knockdown could significantly decrease the protein and mRNA expression of TRPML1 (Figure 5A,B). Further study showed that TRPML1 knockdown reduced the iron-induced secretion of L-ferritin, accompanied by the increased intracellular protein level of L-ferritin in the astrocytes (Figure 5C,D). This led to the hypothesis that TRPML1 knockdown blocked the secretion of L-ferritin, which might be responsible for the accumulation of L-ferritin in the astrocytes. This suggested that TRPML1-mediated trafficking and exocytosis might be involved in the iron-induced secretion of ferritin in astrocytes.

### 3.6. Lentivirus-Mediated Rab27a Gene Knockdown Further Enhanced Ferritin Secretion

**Figure 6:**
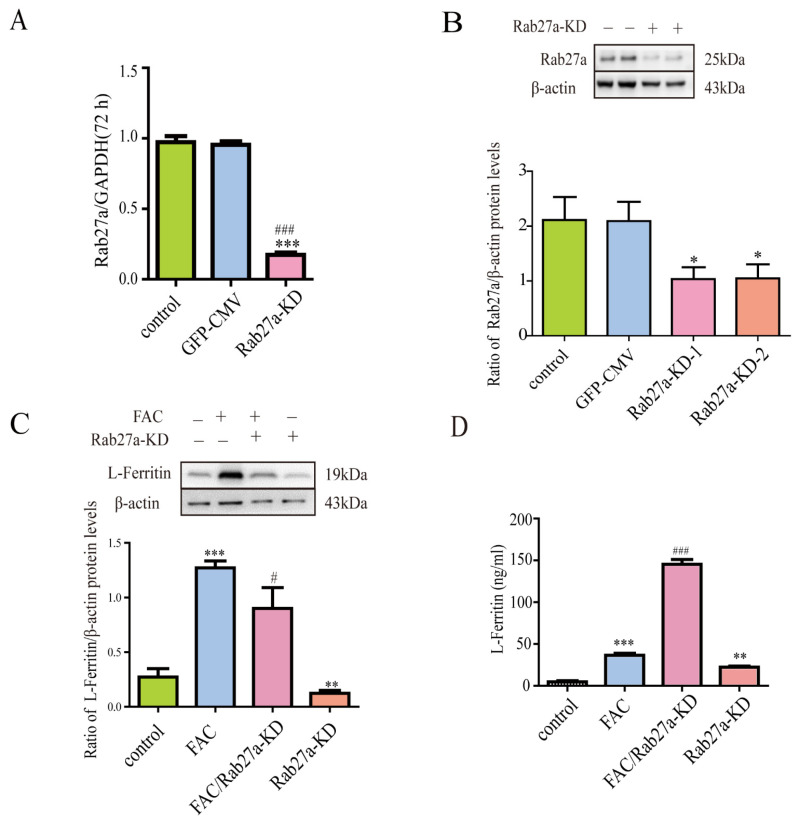
Rab27a knockdown enhanced ferritin secretion. (**A**,**B**) The efficiency of Rab27a knockdown in astrocytes was evaluated by qPCR (**A**) and Western blots (**B**). Data are presented as the mean ± SEM, n = 6, * *p* < 0.05, and *** *p* < 0.001, compared with control; ^###^ *p* < 0.001, compared with GFP-CMV. (**C**,**D**) Rab27a knockdown decreased intracellular protein level of L-ferritin and further enhanced iron-induced L-ferritin secretion. Western blots were used to detect the intracellular protein level of L-ferritin (**C**); ELISA kit was used to detect the extracellular level of L-ferritin (**D**). Data are presented as the mean ± SEM, n = 6, ** *p* < 0.01, and *** *p* < 0.001, compared with control; ^#^ *p* < 0.05 and ^###^ *p* < 0.001, compared with FAC.

Rab27a could regulate vesicle trafficking. To further investigate whether Rab27a was involved in the iron-induced secretion of ferritin from astrocytes, lentivirus-mediated Rab27a knockdown was used in this study. Results showed that Rab27a knockdown significantly decreased the mRNA and protein expression of Rab27a in astrocytes (Figure 6A,B). Further study showed that Rab27a knockdown further enhanced iron-induced L-ferritin secretion and decreased iron-induced L-ferritin protein levels in astrocytes (Figure 6C,D). In addition, our results also showed that Rab27a-ShRNA alone increased L-ferritin secretion.

### 3.7. Secretory Autophagy Protein TRIM16 and sec22b Decreased in Iron-Treated Astrocytes

**Figure 7:**
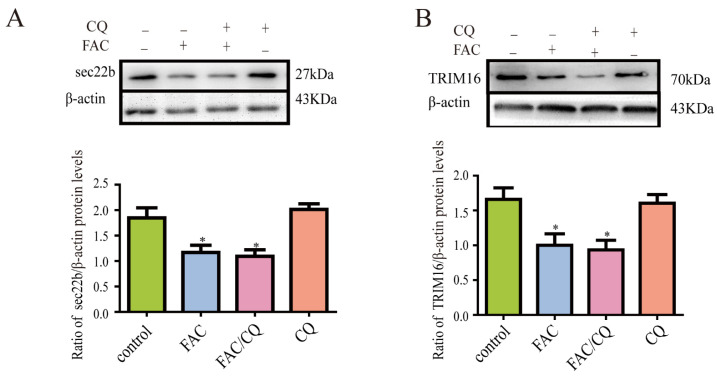
Secretory autophagy protein sec22b and TRIM16 decreased in iron-treated astrocytes. (**A**) Detection of sec22b expression by Western blots after iron treatment or CQ pretreatment in astrocytes. The expression of sec22b decreased after iron treatment, and CQ pretreatment had no effect on the expression of sec22b. (**B**) The protein expression of TRIM16 after iron treatment or CQ pretreatment in astrocytes. Iron induced a decrease in the expression of TRIM16, which could not be inhibited by CQ. Data are presented as the mean ± SEM, n = 8, * *p* < 0.05, compared with control.

It has been reported that secretory autophagy was involved in the secretion of ferritin in cells [41]. Their results showed that TRIM16 and sec22b participated in the process of ferritin secretion. To further investigate the effect of TRIM16-mediated secretory autophagy in iron-induced ferritin secretion, we detected the protein expression of TRIM16 and sec22b in astrocytes after iron treatment. Results showed that the protein expression of TRIM16 and sec22b decreased significantly after iron treatment compared to the control. This indicated that TRIM16 and sec22b might not be involved in the iron-induced increase in ferritin secretion of astrocytes. Further study showed that CQ pretreatment did not change the protein expression levels of sec22b and TRIM16 in iron treated astrocytes, compared with the FAC group (Figure 7A,B).

## 4. Discussion

In the present study, we described the effect of iron treatment on ferritin secretion from astrocytes and elucidated the possible unknown mechanisms underlying this process. We showed that the primary cultured astrocytes do have the ability to secrete ferritin, which is enhanced by iron treatment. Secreted ferritin from iron-treated astrocytes is mainly L-ferritin. Increased ferritin secretion was accompanied by the increased protein expression of ferritin response to iron stimulation. Further study showed that TRPML1-mediated lysosomal exocytosis might be involved in the secretion of ferritin in iron-treated astrocytes. This provides new evidence for the mechanisms underlying ferritin secretion in astrocytes under the condition of high iron levels.

The expression of ferritin is mainly regulated by the interaction of cytoplasmic RNA binding protein iron regulatory proteins (IRPs) with the iron response element (IRE) on ferritin. IRPs can specifically recognize the sequence and spatial structure of the IRE, which is a conserved stem-loop structure in the untranslated area (UTR) of target mRNA [42]. Mutations in the IRE can cause iron disorders and diseases, indicating that IRP/IRE regulation plays an important role in iron homeostasis [43,44,45]. IRPs regulate iron metabolism at the translation level by binding to ferritin mRNA. Under the condition of iron deficiency, IRPs have a high affinity with the IRE in ferritin mRNA, thereby inhibiting its translation and reducing the level of ferritin. In iron-sufficient cells, the binding activity of IRPs to the IRE is reduced, allowing the translation of ferritin mRNA, thereby increasing the levels of ferritin [46]. Consistent with previous studies, we showed that iron treatment increased the protein levels of both L-ferritin and H-ferritin dependent on the IRP/IRE system in astrocytes, which might be responsible for preventing iron-mediated toxicity. Further study showed that the extracellular iron level increased after FAC treatment for 12 h or 24 h, while ferritin secretion increased after FAC treatment for 3–24 h. This suggested that iron-induced ferritin secretion might be responsible for the extracellular iron homeostasis in response to iron treatment.

In our experiment, we observed the secretion of ferritin from astrocytes, which could be enhanced by iron treatment. However, the mechanisms underlying iron-induced ferritin secretion from astrocytes were unclear. The lysosome was previously considered as the end point of endocytosis and was mainly responsible for the degradation of large molecules. It has now been discovered that lysosomes can also act as secretory organelles with specialized mechanisms for regulating protein secretion in certain cell types [47]. Previous study has proved that non-classical lysosomal secretory pathway might be involved in the secretion of ferritin in macrophages due to lack of the N-terminal signal peptide [26]. However, the secretion of ferritin and the underlying mechanisms in astrocytes, especially under iron treatment, were not fully elucidated.

CQ is currently the most widely used lysosomal inhibitor [48], which could inhibit the fusion between autophagosomes and lysosomes [49]. To further investigate the possible mechanisms underlying the secretion of ferritin from astrocytes, CQ was used in this study to detect the role of lysosomes in ferritin secretion. Our results demonstrated that CQ pretreatment could inhibit the increase in ferritin secretion induced by iron treatment in astrocytes. This suggested that the lysosomal pathway might be involved in the secretion of ferritin. In addition, we also selected another inhibitor of autophagy 3-MA to further confirm the possible effect of the lysosomal secretory pathway on iron-induced ferritin secretion. 3-MA could inhibit autophagy by inhibiting the formation of autophagosomes via the inhibition of type III phosphatidylinositol 3-kinases (PI3K) [50]. The results showed that 3-MA also inhibited FAC-induced ferritin secretion. In addition, we also observed the punctate ferritin and colocalization of ferritin with lysosome marker LAMP1 in the presence of iron. This provides further evidence for the presence of ferritin in lysosomes. These results indicated that the secretion of ferritin in astrocytes might be related to the non-classical lysosomal pathway, and specific blocking of the lysosomal pathway had an inhibitory effect on iron-induced ferritin secretion.

Lysosomal exocytosis is a process of secreting lysosomal content by fusion with the plasma membrane, which plays an important role in cellular clearance [51]. The transient receptor potential mucolipin 1 (TRPML1) is a cation channel that exists in the endosomal and lysosomal membranes [52]. It plays a critical role in organellar trafficking, including the fusion of autophagosomes with lysosomes and lysosomal exocytosis [53]. TRPML1 has been demonstrated to mediate lysosomal Ca^2+^ release and Ca^2+^-dependent lysosome trafficking [40]. Activating TRPML1 can promote the release of Ca^2+^ and, subsequently, lysosomal exocytosis. In order to further verify the relationship between the secretion of ferritin and lysosomal exocytosis, we designed and synthesized a lentivirus that can knock down TRPML1 in astrocytes. Then, Western blots and ELISA were used to detect the level of ferritin in astrocytes and outside the cells. Results demonstrated that TRPML1 knockdown reduced the iron-induced secretion of ferritin, accompanied by increased intracellular protein levels of ferritin in astrocytes. This leads to the hypothesis that blocking the secretion of ferritin by TRPML1 knockdown might be responsible for ferritin accumulation in the astrocytes. This suggested that TRPML1-mediated trafficking and lysosomal exocytosis might be involved in the iron-induced secretion of ferritin.

There are a variety of molecules that play a regulatory role in the lysosomal secretory pathway. The Rab-GTPase family is the largest one and is responsible for this process. There are more than 60 subunits of the Rab-GTPase family [54]. Among them, Rab27a is widely distributed, which is related to different intracellular vesicles and organelle trafficking [55,56,57,58,59,60], and participates in vesicle formation [61]. It is reported that Rab27a plays a critical regulatory role in the movement of intracellular vesicles, membrane fusion, and the interaction between vesicles [62]. Rab27a has been demonstrated to play a critical role in the fusion between MVBs and the plasma membrane, and its silencing can reduce the secretion of exosomes. In order to verify whether the secretion of ferritin induced by iron treatment is related to Rab27a, we synthesized a lentivirus that can knock down the Rab27a gene to further detect the effect of Rab27a on ferritin secretion in astrocytes. Results showed that Rab27a knockdown further enhanced iron-induced ferritin secretion and decreased iron-induced ferritin protein levels in astrocytes. In addition, our results also showed that Rab27a knockdown alone increased ferritin secretion. The underlying mechanisms of this effect were not known. This might indicate that ferritin could be exported by multiple pathways. Further study should be conducted to detect the protein level of ferritin in the exosomes of astrocytes after iron treatment and elucidate the possible role of Rab27a in ferritin secretion.

Studies have reported that ferritin might be secreted to the outside of cells through TRIM16-mediated secretory autophagy [28]. Ferritin can be recognized by TRIM16 in specific secretory autophagy, and then TRIM16 interacts with Sec22b to recruit ferritin to the LC3-II isolation membrane for transport [40]. To further verify the possible role of TRIM16-mediated secretory autophagy in ferritin secretion, we detected the protein expression of sec22b and TRIM16 in iron-treated astrocytes. Our experiments confirmed that high-concentration iron decreased the protein expression of both sec22b and TRIM16 in astrocytes, while CQ pretreatment did not change the protein expression of sec22b and TRIM16 significantly compared with the FAC group. This indicated that TRIM16-mediated secretory autophagy might not be involved in the iron-induced increase in ferritin secretion.

## 5. Conclusions

**Figure 8:**
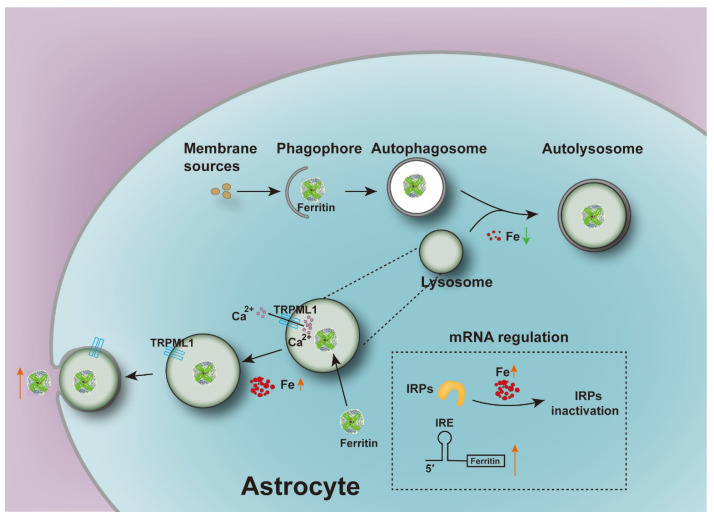
Working model illustrates the mechanism of iron-induced ferritin secretion from astrocytes.

Taken together, our study confirmed that astrocytes could secrete ferritin, which could be enhanced by high iron concentration in primary cultured astrocytes. This might protect DA neurons in the brain against iron-mediated oxidation. In addition, ferritin might be translocated to lysosomes in response to iron treatment in order to protect lysosomes against reactive iron. And, iron treatment also induced ferritin secretion, possibly through TRPML1-mediated exocytosis from the lysosomes of astrocytes (Figure 8). Our previous study has shown that ferritin released by astrocytes might be involved in the protection of MES23.5 dopaminergic cells against 1-methyl-4-phenylpyridinium ion (MPP^+^)-induced neurotoxicity and ferroptosis [28]. In addition, we also demonstrated that apoferritin exerted a neuroprotective effect against MPTP-induced PD mice model by regulating brain iron metabolism [63]. Therefore, targeting astrocytes to regulate ferritin secretion and iron homeostasis might provide promising therapeutic approaches for PD.

When intracellular iron levels decrease, ferritin is delivered to lysosomes for autophagic degradation. When intracellular iron levels increase, the binding activity of IRPs to the IRE is reduced, allowing the translation of ferritin mRNA, thereby increasing the expression of ferritin. Meanwhile, iron increases the secretion of ferritin, possibly via TRPML1-mediated exocytosis.

## Data Availability

All data generated or analyzed for the study are included in this published article.
